# Advances in lung cancer screening and early detection

**DOI:** 10.20892/j.issn.2095-3941.2021.0690

**Published:** 2022-05-11

**Authors:** Caichen Li, Huiting Wang, Yu Jiang, Wenhai Fu, Xiwen Liu, Ran Zhong, Bo Cheng, Feng Zhu, Yang Xiang, Jianxing He, Wenhua Liang

**Affiliations:** 1 Department of Thoracic Oncology and Surgery, the First Affiliated Hospital of Guangzhou Medical University, China National Center for Respiratory Medicine, China State Key Laboratory of Respiratory Disease & National Clinical Research Center for Respiratory Disease, Guangzhou Institute of Respiratory Health, Guangzhou 510120, China; 2 Dongguan Affiliated Hospital of Southern Medical University, Dongguan People Hospital, Dongguan 523059, China; 3 Department of Internal Medicine, Detroit Medical Center Sinai-Grace Hospital, Detroit, Michigan 48235, USA; 4 Department of Thoracic Surgery, Nanfang Hospital of Southern Medical University, Guangzhou 510515, China; 5 Department of Oncology, the First People’s Hospital of Zhaoqing, Zhaoqing 526020, China

**Keywords:** Lung cancer, screening, low-dose CT, early detection, strategies, biomarkers

## Abstract

Lung cancer is associated with a heavy cancer-related burden in terms of patients’ physical and mental health worldwide. Two randomized controlled trials, the US-National Lung Screening Trial (NLST) and Nederlands-Leuvens Longkanker Screenings Onderzoek (NELSON), indicated that low-dose CT (LDCT) screening results in a statistically significant decrease in mortality in patients with lung cancer, LDCT has become the standard approach for lung cancer screening. However, many issues in lung cancer screening remain unresolved, such as the screening criteria, high false-positive rate, and radiation exposure. This review first summarizes recent studies on lung cancer screening from the US, Europe, and Asia, and discusses risk-based selection for screening and the related issues. Second, an overview of novel techniques for the differential diagnosis of pulmonary nodules, including artificial intelligence and molecular biomarker-based screening, is presented. Third, current explorations of strategies for suspected malignancy are summarized. Overall, this review aims to help clinicians understand recent progress in lung cancer screening and alleviate the burden of lung cancer.

## Introduction

Lung cancer is the second most common cancer and the leading cause of cancer-related mortality worldwide, with an estimated 2.2 million new cases and 1.8 million deaths in 2020, thus imposing severe social and economic burdens^1^. According to histological type, lung cancer can be divided into non-small cell lung cancer (NSCLC) and small cell lung cancer. NSCLC accounts for approximately 85% of all lung cancers, and adenocarcinoma and squamous cell carcinoma are the main histopathologic subtypes^2^. Despite advances in surgery, radiotherapy, chemotherapy, molecularly targeted therapy, and immunotherapy, the average 5-year survival rate for lung cancer is only 19%^3^.

The 5-year survival rate for early-stage lung cancer is greater than that for advanced-stage lung cancer. Patients with stage IA lung cancer have a high 5-year survival rate exceeding 75%^4^. As cancer progresses, the long-term survival rate dramatically decreases. For instance, the average 5-year survival rate for stage IV lung cancer is less than 10%^4^. Given the frequent absence of symptoms before locally advanced or metastatic deposit, and the aggressive and heterogeneous biological characteristics of the disease, clinical intervention is more effective in earlier stages of lung cancer. Advanced lung cancer is often accompanied by compressing, invading, high metastatic tendency, or paraneoplastic syndromes, thus resulting in poor survival prognosis^5^. Furthermore, advanced lung cancer may correlate with higher tumor mutation burden and genetic instability^6^, and transformed cancer cells may use immune evasion approaches to create an immunosuppressive tumor microenvironment and escape anti-tumor immunosurveillance^7^. Consequently, the tumor doubling time is shortened, thus leading to tumor progression and unsatisfactory treatment responses^7^.

The 5-year survival rate of early stage lung cancer can be increased by 20%–30% through radical surgery, but approximately 85% of patients with lung cancer are diagnosed in advanced stages^4^. Although the available data on patients with small cell lung cancer are limited, the prognosis appears to be better when the disease is diagnosed at an early stage^8^. Therefore, early screening and diagnosis are the most effective approaches for improving long-term survival and are being actively explored. This review presents an overview of the current evidence and techniques for lung cancer screening, and explores feasible strategies for the early detection and diagnosis of lung cancer. We focus on the evidence for screening with low-dose computed tomography (LDCT) worldwide, the entry criteria for screening, and advances in differential diagnosis and treatment strategies for suspected malignancies.

### The evolution of lung cancer screening approaches

Lung cancer screening began in the 1960s, with an aim to diagnose early-stage lung cancer and decrease lung cancer-related mortality. The most common methods used from the 1960s to the 1990s were chest X-ray and sputum cytology. The Mayo clinical trial was the first randomized controlled trial (RCT) focusing on high-risk populations (men 45 years or older who smoked at least 20 cigarettes per day in the prior year). All participants underwent a baseline chest X-ray and sputum cytologic examination, followed by chest X-ray and sputum cytology every 4 months in the screening arm, and yearly chest X-ray and sputum cytology in the control arm. Although more cancer cases were found in the screening groups, no difference in lung cancer deaths was observed between groups^9^. Subsequently, a series of studies examined chest X-ray screening alone or in combination with sputum cytology, but did not find a mortality benefit^10–12^. Given the relatively small sizes of the above trials, a lung study within the Prostate, Lung, Colorectal, and Ovarian (PLCO) cancer screening trial was performed to compare annual chest radiographic screening with usual care. Importantly, the PLCO trial screened for multiple cancers, and smoking history was not an inclusion criterion. At the 13-year follow-up, annual chest X-ray screening, compared with usual care, was not found to decrease lung cancer mortality^13,14^.

Because of the low sensitivity of chest X-rays, the detection of lung cancer by computed tomography was first described and received substantial attention in the 1990s, and has subsequently been shown to be superior to chest X-rays in observational studies^15,16^. LDCT is a computed tomography technique that uses X-rays to create internal body images. The effective radiation dose in chest LDCT is approximately 1.5 mSv, which is 15 times higher than that of traditional chest radiography but less than one-quarter that of conventional chest CT^17^. LDCT has been demonstrated to be the only efficient and promising approach for lung cancer screening, on the basis of 2 independent, sufficiently powered RCTs^18,19^.

### Screening with LDCT

#### Evidence from the US and Europe

In 2011, results from the US-based National Lung Screening Trial (NLST) indicated a 20% decrease in lung cancer-related mortality after a median follow-up of 6.5 years in patients undergoing annual LDCT screening compared with scanning by radiography at the same frequency for 3 years^19^. Notably, a relative decrease of 6.7% (95% CI 1.2–13.6, *P* = 0.02) in all-cause mortality was also observed in the LDCT group^19^. In addition, several randomized trials of LDCT screening have been performed in Europe, with different recruitment strategies and numbers of screening rounds (**Table 1**). The only individual trial with a sufficient sample size to demonstrate a lung cancer mortality decrease is the Dutch–Belgian lung-cancer screening trial (NELSON)^18^. In 2020, Koning et al.^18^ reported a rate of death due to lung cancer in men that was 24% lower in the screening arm than the control arm after 10 years. A meta-analysis of 7 trials, enrolling more than 84,000 patients with a history of smoking more than 15 pack-years, has indicated a cumulative lower lung cancer mortality in patients scanned with LDCT, with a risk ratio of 0.83 and a nonsignificant decrease in all-cause mortality of 4% with respect to other interventions^31^. This evidence suggests that people with a history of smoking benefit from screening. Hence, chest LDCT has been recommended as the standard screening approach for lung cancer in asymptomatic populations with high risk, in accordance with the National Comprehensive Cancer Network (NCCN), American Cancer Society (ACS), and US Preventive Services Task Force (USPSTF)^32–34^.

**Table 1:** Selected lung cancer screening studies

Study	Interventions	Overall follow-up, years	Participants, *n*	Inclusion criteria	Primary outcome
**NLST** ^19,20^	LDCT *vs.* CXR	7.4	53,454	Age 55–75 years, ≥30 pack-years, <10 years ex-smoker	LDCT decreased lung cancer-related mortality (*HR* = 0.80; *P* < 0.004)
**NELSON** ^18,21,22^	LDCT *vs.* no intervention	10	15,789	Age 55–75 years, ≥15 pack-years, <10 years ex-smoker	LDCT decreased lung cancer-related mortality (*HR* = 0.76; 95% CI 0.62-0.94 in men)
**DANTE** ^23,24^	LDCT *vs.* no intervention	8.35 (median)	2,811	Age 60–74 years, ≥20 pack-years, <10 years ex-smoker	A non-significant decrease was observed in lung cancer-related mortality (*HR* = 0.99)
**DLCST** ^22,25,26^	LDCT *vs.* CXR	10	4,104	Age 50–70 years, ≥20 pack-years, <10 years ex-smoker	A non-significant decrease was observed in lung cancer-related mortality (*HR* = 1.03)
**MILD** ^27,28^	LDCT *vs.* no intervention	10	4,099	Age ≥49 years, ≥20 pack-years, <15 years ex-smoker	LDCT decreased cumulative risk of 10-year lung cancer-related mortality (*HR* = 0.61; *P* = 0.02)
**LUSI** ^29^	LDCT *vs.* no intervention	8.8 (median)	4,052	Age 50–69 years, ≥15 pack-years, <10 years ex-smoker	LDCT decreased lung cancer-related mortality only in women (*HR* = 0.31; *P* = 0.04)
**UKLS** ^59,169^	LDCT *vs.* no intervention	10	4,055	Age 50–75 years, LLP_v2_-defined 5-year lung cancer risk ≥5%	67% of stage I lung cancers were detected in the screening arm; mortality results to be published
**ITALUNG** ^22,165,170,171^	LDCT *vs.* no intervention	8.5 (median)	3,206	Age 55–69 years, ≥20 pack-years, <10 years ex-smoker	A non-significant decrease was observed in lung cancer-related mortality (*HR* = 0.7)
**DEPISCAN** ^30^	LDCT *vs.* CXR	4	765	Age 50–75 years, ≥15 pack-years, <15 years ex-smoker	LDCT enabled the detection of more lung cancers than CXR (8 *vs.* 1)
**Beijing I-ELCAP cohort** ^38^	LDCT	5	4,690	Age ≥40 years, without cancer history within 5 years	The diagnosis rate of lung cancer by LDCT was 76.0%, and most of the cancers were detected in an early stage
**Shanghai ChestHosp RCT** ^41^	LDCT *vs.* usual care	2	6,717	Age 45–70 years, ≥20 pack-years, <15 years ex-smoker	LDCT led to a 74.1% increase in detection of early-stage lung cancer
**Shanghai CancerHosp cohort** ^172^	LDCT	1	11,332	Age 50-80 years, ≥20 pack-years, <5 years ex-smoker	LDCT improved the early diagnosis rate of lung cancer in both smokers and nonsmokers
**Shanghai-ChangzhengHosp cohort** ^43^	LDCT	2	14,506	Age >35 years, asymptomatic participants	The incidental detection rate for stage I lung cancer was 0.97%
**Anti-Lung Cancer Association Project** ^173^	LDCT *vs.* CXR *vs.* sputum cytology	7	1,611	Age ≥40 years, asymptomatic participants	The proportions of positive tests were 9.1%, 2.6%, and 0.7% with low-dose helical CT, chest X-ray, and sputum cytology
**Mass screening for lung cancer with mobile spiral computed tomography scanner** ^15,174^	LDCT	5	5,438	Age 40–74 years	The lung-cancer detection rate with CT was 0.48%, significantly higher than the 0.03%–0.05% for standard mass assessments previously performed in the same geographic area
**Hitachi Employee’s Health Insurance Group program** ^175^	LDCT	3	7,956	Age 50–69 years	The prevalence was 0.44% among all participants from baseline, and 0.07% from repeated screening
**Hitachi lung cancer screening program** ^44^	LDCT *vs.* CXR	6	17,935	Aged 50–64 years, <30 pack-years	LDCT decreased lung cancer-related mortality (*HR* = 0.76; *P* < 0.001)
**Samsung Medical Center program** ^176^	LDCT	5	6,406	Age ≥45 years, asymptomatic participants	LDCT helped detect early-stage lung cancer in an asymptomatic Korean population, with a detection rate of 0.36%
**K-LUCAS Project** ^177^	LDCT	1	256	Age 55–75 years, ≥30 pack-years, <10 years ex-smoker	7.4% of participants exhibited positive findings

CI, confidence interval; CXR, chest X-ray; DANTE, Detection and screening of early lung cancer with novel imaging technology; DEPISCAN, french randomized pilot trial of lung cancer screening comparing low-dose CT scan and chest X-ray; DLCST, Danish lung cancer screening trial; HR, hazard ratio; ITALUNG, italian lung cancer screening trial; LDCT, low-dose computed tomography; LUSI, german lung cancer screening intervention; K-LUCAS, korean lung cancer screening; MILD, multi-centric Italian lung detection; NELSON, Dutch-Belgian randomized lung cancer screening trial; NLST, national lung screening trial; UKLS, UK lung cancer screening trial.

In addition, analysis of lung cancer incidence and mortality through the US Surveillance, Epidemiology, and End Results (SEER) and the Global Burden of Disease (GBD) database in recent years has indicated that the proportion of stage I in lung cancer in the US significantly increased after the broad clinical introduction of LDCT screening in 2002, when the NLST trial started^19^. In 2011, after LDCT was approved for lung cancer screening, the lung cancer-related mortality for stages I to III has undergone an average annual decline exceeding 10%, thereby indicating the value of CT screening in increasing overall lung cancer survival at the population level^35^.

#### Evidence from Asia

Lung cancer is more common in women and non-smokers in Asia than in Europe and the US^36^, thus indicating that the Asian population may require different lung cancer screening guidelines from those in Western countries. In China, prior studies have mainly included occupational populations for lung cancer screening with chest X-ray and/or sputum cytology^37^. One of the earliest lung cancer screening programs applying LDCT was launched in 2006, involving 4,690 asymptomatic participants in Beijing^38^. The International Early Lung Cancer Action Program (I-ELCAP) algorithm was used to follow up on initial abnormal LDCT scan findings, and 19 of the 25 cases were in stage I. These preliminary results suggested that LDCT can detect lung cancer in early stages^38^. Subsequently, national screening projects for both rural [rural China screening program (RuraCSP)] and urban [cancer Screening program in urban China (CanSPUC)] areas were launched in 2009 and 2012, respectively. However, because LDCT screening trials in China began relatively later than those in other countries, Netherlands-China Big-3 (NELCIN-B3) screening was established^39,40^ to investigate the effectiveness of LDCT screening according to a volume-based protocol in the Chinese population. The results will be available in the near future.

Several screening trials funded by the municipal government, such as Shanghai, Guangzhou, in China have reported baseline results. In Shanghai, an LDCT-based lung cancer screening study including 6,717 participants has investigated whether LDCT screening might improve the early-stage lung cancer detection rate in Chinese patients with high-risk profiles^41^. In the 2-year follow-up period, lung cancer was found in 1.5% of patients who received LDCT screening, and LDCT, compared with usual care, resulted in 74.1% greater detection of early stage lung cancer^41^. In addition, according to a modeling study, LDCT-based screening of high-risk people in Chinese urban areas resulted in 17.2% and 24.2% lower lung cancer-related mortality than did chest radiography-based screening and no screening, respectively^42^. Another LDCT-based screening study including 14,506 participants in Shanghai has reported a lung cancer detection rate of 1.23%, with a 0.97% incidental detection rate of stage I lung cancer^43^, thus suggesting the potential benefits of decreased lung cancer morbidity and mortality. Of note, this cohort focused on asymptomatic residents with a minimum age of 35, regardless of risk factors^43^.

In Japan, a population-based cohort study was performed in the Hitachi region, including participants 50–64 years of age with a smoking history of less than 30 pack-years^44^. The study demonstrated a lung cancer mortality benefit 24% (0.76, 95% CI 0.67–0.86, *P* < 0.001) above the average for all of Japan^44,45^. A significant decrease in standardized mortality was observed among screened female participants (0.74, 95% CI 0.56–0.97)^45^. Notably, more than 90% of female participants were non-smokers, thus indicating that LDCT-based screening can decrease lung cancer-related mortality in non-smokers and smokers^45^. In Korea, the National Korean Lung Cancer Screening Project (K-LUCAS), a single-arm trial of 256 high-risk patients, used the same inclusion criteria as the NLST^46^. With the lung imaging reporting and data system (Lung-RADS) algorithm, 10 nodules were classified as grade 3; 9 nodules were classified as grade 4; and 1 nodule was finally diagnosed as lung cancer^46^.

Although LDCT-based lung cancer screening is increasing in Asia, the entry criteria for trials across countries have not used concordant definitions of high risk. Given the geographic and lifestyle differences across Asia, specific high-risk criteria must be proposed for different areas to improve the benefits of screening in the future. Moreover, implementing individual risk-based screening remains a major challenge that must be addressed.

#### Risk-based selection for screening

Previous epidemiological studies have indicated that a history of smoking^47,48^, second-hand smoke exposure, or environmental oil smoke inhalation^49,50^; history of occupational carcinogen exposure^51^; family history of lung cancer^52,53^; history of cancer^54^; and history of lung diseases (chronic obstructive pulmonary disease, tuberculosis, or pulmonary fibrosis)^55–57^ are closely associated with the incidence of lung cancer. However, Bach et al.^58^ have indicated that LDCT screening might result in a 3-fold increase in lung cancer diagnosis and a 10-fold increase in lung cancer-related surgery accompanied by potential psychological and physical burden. To balance the benefits and potential harms with screening, the high-risk population must be precisely defined within the general population. Most screening trials in the US and Europe have defined the entry criteria for screening by a combination of age and smoking exposure^32^. The age eligibility has ranged from 40 years of age to no limit in most screening trials. The initial screening age of 55 recommended by NCCN, ACS, and USPSTF has been based primarily on the eligibility criteria for NLST since 2011^19^. The UK Lung Cancer Screening (UKLS) teams have suggested that screening eligibility in people at least 58 years of age would be reasonable, owing to the relatively sharp increase in lung cancer incidence at this age (1%–4.3%)^59^. However, because the appropriate age range for lung cancer screening remains a matter of debate in Asia, the starting screening age in trials conducted in Asia varies among countries (**Table 1**).

Several risk prediction models including multiple risk factors, such as smoking and age, have been developed to identify high-risk groups in recent years. For instance, the Liverpool Lung Project model (LLP)^60^, Pan-Canadian Early Detection of Lung Cancer (PanCan)^61^, and PLCO_2012_^62^ models have shown good diagnostic accuracy on the basis of patients’ clinical characteristics, epidemiology, and social risk factors. The LLP model was developed from a case-control study in Liverpool, considering age, male sex, smoking duration, history of COPD, past diagnosis of malignant tumors, and family history of early-onset lung cancer (<60 years) as significant risk factors^60^. The UKLS RCT has applied the LLP model as an entry criterion and identified more lung cancer cases than NLST (1.7% *vs.* 1.03%) at baseline^59^. The PanCan model has used age, smoking duration, pack-years, family history of lung cancer, education level, body-mass index, chest X-ray in the prior 3 years, and history of chronic obstructive pulmonary disease as risk factors^61^. A retrospective study has indicated that the cumulative incidence according to the PanCan model is significantly higher than that in NLST^63^. The PLCO_2012_ model includes age, race, socioeconomic status, BMI, history of COPD, history of cancer, family history of lung cancer, smoking status, and smoking cessation^62^. This model based on individual risk also has shown better diagnostic accuracy than entry criteria based on a combination of age and smoking exposure. Notably, the LLP model used in UKLS has been the only risk model used to select people in RCTs of lung cancer screening. In China, Guo et al.^64^ have reported a risk model for selecting high-risk populations that includes age, sex, smoking, history of tuberculosis, and history of emphysema, on the basis of data from CanSPUC; the C-index of the model has been found to be 0.741 in the validation set for 1-year lung cancer risk. Interestingly, another risk model consisting of 6 variables (age, sex, education level, tuberculosis, hyperlipidemia, and family history of lung cancer) for non-smokers has shown moderate predictive discrimination, with areas under the curve of 0.668, 0.678, and 0.685 for 1-, 3-, and 5-year lung cancer risk, respectively^40^. Risk prediction models may facilitate efficiency and decision-making in key steps in lung cancer screening. Nevertheless, because current evidence is insufficient to determine which models are most clinically beneficial, further clinical studies are needed to validate theoretical prediction.

To decrease the rate of missed lung cancer detection, Li et al.^65^ have aimed to identify new lung cancer risk factors. A population-level analysis in 110,000 participants from 26 lung cancer screening studies has indicated that the proportion of stage I lung cancer among all lung cancer cases decreases with age, and is higher with screening at ages of 40 or 45 than 50 or 55. The findings suggest that a substantial number of cancer foci already exist in people between 40 and 50 years of age^65^. In addition, Peng et al.^66^ have performed a comprehensive study combining meta-analysis with Mendelian randomization to determine risk factors causally related to the incidence of lung cancer. The study has revealed that systemic lupus erythematosus is associated with an elevated risk of lung cancer. Meanwhile, a mass screening targeted high-risk and non-high-risk patients over the age of 40 has been performed in Guangzhou, China (NCT04938804), to investigate lung cancer risk factors. In summary, participants at high risk may be identified with an individual risk prediction model combining epidemiological data and biomarkers (discussed below) to achieve maximum benefits in the future.

### Advances in differential diagnosis of pulmonary nodules

The developments in lung cancer screening and LDCT have increased the rate of detection of small pulmonary nodules^67^. However, malignant nodules usually lack specific manifestations in LDCT. Differentiating lung cancer (such as minimally invasive adenocarcinoma or minimally invasive adenocarcinoma) from atypical adenomatous hyperplasia and other benign nodules is challenging. Therefore, the ability to accurately detect and diagnose malignant nodules will decrease the cost of additional examinations and the risk of missing malignant nodules, thus enabling early lung cancer treatment. Furthermore, because of the potential harms of LDCT (as discussed previously), an urgent clinical need exists to develop non-invasive approaches to improve screening accuracy for high-risk patients.

#### Artificial intelligence (AI)-based screening

AI has played a crucial role in malignant nodule screening and computer-aided diagnosis with advances in medical imaging and the development of deep neural network learning methods. Ding et al.^68^ have developed a modified Faster R-CNN (based on Region-Based Convolutional Neural Network) for the detection of malignant pulmonary nodules with a true positive rate of 94.60%. Nasrullah et al.^69^ have designed a Faster R-CNN with customized mixed link network (CMixNet) and U-Net-like encoder-decoder architecture for nodule detection. This system yields an average of 8 false positives per scan and a true positive rate of 94.21%. Khosravan et al.^70^ have applied a 3D CNN called S4ND based on the Single-Shot Single-Scale lung Nodule Detection system to detect lung nodules without further processing, with a true positive rate of 95.20%. Another team has designed Deep 3D Dual Path Nets (3D DPN26) with 3D Faster R-CNN and achieved a true positive rate of 95.80% with high sensitivity^71^.

Automatically detected lung nodules must be diagnosed to determine whether they are benign or malignant. Ren et al.^72^ have developed a manifold regularized classification deep neural network (MRC-DNN) to automatically determine benign malignancy with an accuracy of 90.00%. Hussein et al.^73^ have used a standard 3D CNN architecture to evaluate the characteristics of different nodules, including calcification, lobulation, sphericity, speculation, margin, and texture, and then generate the malignant score of the nodules with an accuracy of 91.26%. Kang et al.^74^ have designed 3D multi-view CNN (MV-CNN) based on 3D Inception and 3D Inception-ResNet architectures. This system allows nodules to be divided into benign and malignant groups. Experiments performed on an LIDC-IDRI dataset with 10-fold cross-validation have indicated an accuracy of 95.25%^74^. At present, the problems of automatic detection and diagnosis of lung nodules have not been completely solved, and include overfitting, a lack of interpretability, and insufficient annotation data. In addition, because this is an emerging field, these models still lack standardization approaches, and their comparability and replicability are also worthy of consideration. Fortunately, the demand for early detection of lung cancer has promoted the development of AI, which is expected to make lung cancer screening more accurate and commonly used.

#### Biomarkers for screening

A useful biomarker in lung cancer screening should enable clinical decision-making, aid in early diagnosis, and improve risk stratification. The application of biomarkers to predict the diagnosis of malignant nodules must not increase the number of diagnostic procedures for benign nodules or delay the treatment for malignancy^75^. Blood-derived biomarkers may serve as primary and metastatic lesions to better characterize tumor heterogeneity; therefore, blood has been the preferred source of biomarker candidates. In addition, bronchial lavage, exhaled breath, saliva, and urine are potential sources of biomarkers for lung and other respiratory tract cancers. Of note, most biomarkers are currently in development and clinical validation, and few currently available biomarkers have been demonstrated to decrease lung cancer-related mortality.

#### ctDNA and ctDNA methylation

To date, technologies such as next-generation sequencing technology (NGS) have markedly decreased the limits of detection and promoted the clinical utility of ctDNA screening^76,77^. Although its levels are low in early-stage lung cancer, ctDNA can be detected before treatment in most patients^78^. Newman et al.^79^ have developed a method based on CAPP-Seq for NSCLC, with a sensitivity of 100% for patients with stage II–IV cancer and 50% for patients with stage I cancer. Through targeted error correction sequencing (TEC-Seq) using massively parallel sequencing, Phallen et al.^80^ have reported a sensitivity of 59% for patients with stage I or II lung cancer. However, these detection methods might decrease the specificity, owing to the presence of mutated genes in noncancerous tissue. Recently, a machine learning approach called DELFI has been used to detect abnormalities in cfDNA through genome-wide analysis of fragmentation patterns for various cancer types^81^. DELFI has detected 94% of patients with cancer across stages and subtypes, with a sensitivity of 91% for stage I or II, and 96% for stage III or IV, and a high specificity of 80%^82^.

Cancer is often characterized by hypermethylation of promoters at specific CpG sites associated with tumor suppressor genes^83^. The GRAIL method combines bisulfite sequencing and machine learning to read methylated DNA sequences and has shown high specificity across many cancers^84^. Observational cohort studies and an interventional study have assessed the performance of targeted methylation analysis of cfDNA to detect and localize multiple cancer types^85–87^. The preliminary trials have indicated a specificity of 99.3% (95% CI, 98.3%–99.8%) across all stages^88^. The stage I–III sensitivity has been found to be 67.3% (95% CI, 60.7%–73.3%) in a pre-specified set of 12 cancer types^88^. The recently published results of the Circulating Cell-free Genome Atlas Study (CCGA) have indicated a specificity for cancer signal detection of 99.5% (95% CI, 99.0%–99.8%)^89^. The overall sensitivity for cancer signal detection was 51.5% (49.6%–53.3%), and the stage I–III sensitivity was 67.6% (64.4%–70.6%) in 12 pre-specified cancers^89^. Lung cancer data are expected to be released in the future. In China, Liang et al.^90^ have developed a diagnostic model based on targeted DNA methylation sequencing, which has 80% accuracy in the discrimination of benign and malignant lung nodules at different stages of cancer, outperforming the PET-CT, Mayo Clinic, and Veterans Affairs prediction models^91^. Selected clinical trials using liquid biopsy for cancer screening are summarized in **Table 2**.

**Table 2:** Clinical trials evaluating the potential applications of ctDNA in lung cancer screening

Identifier	Methods	Initiator	Study type	Estimated enrollment, *n*	Observational model	Time perspective	Status/results
NCT04814407	ctDNA methylation EMS-ddPCR scLSM-FACS	Taiwan University Hospital	Observational	900	Cohort	Prospective	Recruiting; estimated study completion date is December 31, 2027
NCT04712877	Genetic mutations NGS	Lung Cancer Research Foundation	Observational	1,000	Cohort	Prospective	Not yet recruiting; estimated study completion date is November 1, 2023
NCT03651986	ctDNA methylation NGS	AnchorDx Medical Co., Ltd.	Observational	10,560	Cohort	Prospective	Recruiting; estimated study completion date is June 2023
NCT04253509	ctDNA methylation sequencing	Samsung Medical Center	Observational	280	Cohort	Prospective	Recruiting; estimated study completion date is February 2022
NCT03685669	ctDNA methylation biomarkers	Shanghai Chest Hospital	Observational	300	Cohort	Cross-Sectional	Recruiting; estimated study completion date is December 2019
NCT04698681	Genetic mutations NGS	Calithera Biosciences. Inc	Interventional Clinical Trial	200	Single group assignment	Prospective	Active, not recruiting; estimated study completion date is May 2022
NCT03181490	ctDNA methylation, high-throughput bisulfite DNA sequencing, NGS	The First Affiliated Hospital of Guangzhou Medical University	Observational	1,490	Cohort	Cross-Sectional	Completed; detected early-stage lung cancer and differentiated lung cancers from benign pulmonary nodules
NCT02612350	ctDNA genetic mutations	Pathway Genomics	Observational	1,106	Cohort	Prospective	Completed; provided a new way to investigate screening utility

ctDNA, circulating tumor DNA; EMS-ddPCR, enriched methylation-specific droplet digital PCR; scLSM-FACS, single-cell, locus-specific methylation detection.

#### Autoantibodies

Tumor-associated antigens are usually abnormal proteins expressed as a result of genetic changes in tumor cells or intracellular proteins after necrosis or apoptosis^92,93^. Through an early amplification signal in auto-antibody levels produced during the immune sensing phase, early-stage lung cancer can be detected even in vessel-free tumors^94^. In the Mayo Lung Screening Trial and Kentucky Lung Screening Trial, Trudgen et al.^95^ have demonstrated that antibodies can identify 50% of occult cancers as early as 5 years before diagnosis.

The EarlyCDT-Lung trial developed a panel to measure autoantibodies to 7 cancer-associated antigens (p53, NY-ESO-1, CAGE, GBU4-5, SOX2, HuD, and MAGE A4)^94^ with a sensitivity of 41% in early stages^96^. The UK NIH has conducted an RCT called Early Diagnosis of Lung Cancer Scotland (ECLS), including 12,208 participants at high risk of developing lung cancer^97^. The intervention arm received the EarlyCDT-Lung test followed by LDCT scanning every 6-months for up to 2 years if positive or received standard clinical care as a control if negative. After 2 years, the hazard ratio for stage III/IV presentation was 0.64 (95% CI 0.41–0.99)^97^. In the German Lung Cancer Screening Intervention Trial (LUSI), EarlyCDT-Lung showed similar effectiveness^98^. Through use of different combinations of biomarkers, autoantibodies may achieve a balance of sensitivity and specificity^99–102^. Because of significant differences in genetic variation among patients with lung cancer in Europe and Asia, a 7-AAB panel has been developed and approved in China, with a sensitivity of 59%–64% in NSCLC patients^103^. Because the autoantibody test’s relatively low sensitivity suggests that it is not useful when performed alone for lung cancer diagnosis, it must be combined with another test. When combined with CT or miRNA, the 7-AAB panel has been found to improve the diagnosis of lung cancer and nodules^104–106^, but to be accompanied by a high number of false-positive results^107^. This finding might be due to mutations from normal somatic cells inducing immunity and autoantibody production^108^.

#### Circulating tumor cells (CTCs)

CTCs are active tumor cells in the peripheral blood originating from the primary tumor or metastatic deposits. Even early-stage primary tumors can shed CTCs throughout their development, thus providing a sensitive method for tumor screening^109^. Li et al.^110^ have found that CTC frequencies vary between patients with lung cancer and healthy control volunteers or patients with benign lung disease, and have reported an area under the receiver operating characteristic curve in the control group of 0.846 (95% CI 0.796–0.887, *P* < 0.001). The positivity rate of CTC in patients with lung cancer was 68.29% when the CTC cutoff value was 2^110^. Wei et al.^111^ have found that the numbers of CTCs in patients with NSCLC vary according to the cancer stages and genetic mutations. A prospective study called the AIR Project was conducted in 21 French university centers to determine the value of CTCs in the early detection of lung cancer. Approximately 600 participants underwent yearly LDCT screening and peripheral blood sampling for CTC detection for 3 years plus a 1-year follow-up^112^. The preliminary results indicated that when both CNHC-malignant and CNHC-uncertain were considered positive results, the sensitivity and specificity of CTCs as a biomarker for lung cancer detection were 26.3% (95% CI 11.8–48.8) and 96.2% (95% CI 94.4–97.5) at baseline^113^. Furthermore, a meta-analysis of 21 studies with 3,997 participants has assessed the overall diagnostic accuracy and reported a pooled sensitivity and specificity of 0.72 (95% CI 0.65–0.79) and 0.96 (95% CI 0.91–0.98), thus indicating that CTCs also perform well in the diagnosis of lung cancer^114^.

Several studies have analyzed the feasibility of combining LDCT screening with CTC, aiming to compensate for the technical shortcomings of LDCT alone. One study has applied CTC testing in 32 patients with ground-glass nodules selected on the basis of LDCT screening and has identified cancer-related gene mutations in positive patients^99^. Another study has indicated that CTC numbers differ among high-risk LDCT screened patients, patients with NSCLC, and healthy people^115^. However, validation of CTCs in a large-scale prospective trial has been hindered by the lack of a standardized test, thus making the results difficult to compare^116^. Although CTC testing has become more widely used in early lung cancer screening, owing to technological innovations, this method is mainly used to predict metastasis and prognosis, and perform drug modeling^117–120^.

#### microRNAs (miRNAs)

miRNAs are short, non-coding, stable RNA sequences that regulate gene expression post-transcriptionally. Tumor-secreted miRNAs are detectable in the circulating blood^121^. Wang et al.^122^ have reported a pooled sensitivity and specificity of 0.75 and 0.7 for of miRNAs as biomarkers for NSCLC detection. The miR-test is a serum-based miRNA test that measures a signature of 13 miRNAs^123^. A large-scale validation study in more than 1,000 high-risk patients has reported an overall accuracy, sensitivity, specificity, and area under the curve of the miR-test of 74.9% (95% CI 72.2%–77.6%), 77.8% (95% CI 64.2%–91.4%), 74.8% (95% CI 72.1%–77.5%), and 0.85 (95% CI 0.78–0.92), respectively^123^. Another test, micro-RNA signature classifier (MSC), is a plasma-based miRNA test that categorizes patients into low, intermediate, or high risk of disease on the basis of pre-defined positivity for 24 miRNA expression ratios^124^, with a sensitivity of 87% and specificity of 81% across both arms, and a sensitivity of 88% and specificity of 80% in the LDCT arm^124^. Furthermore, Fehlmann et al.^125^, in Germany, have used genome-wide microRNA profiles from blood samples to identify patients with lung cancer. This multicenter cohort study in 3,102 patients from case-control and cohort studies has reported 91.4% accuracy, 82.8% sensitivity, and 93.5% specificity^125^. Nevertheless, application of this method in clinical practice faces many barriers posed by the technical issues remaining to be solved. For instance, detailed validation of the pre-analytical steps affecting miRNA detection and quantification is critical. A valid method for the normalization results is greatly needed^126^. Moreover, circulating miRNAs do not show specificity for a type of cancer—a matter of particular concern.

#### Circulating protein profiling

Previous studies have shown that antigen 125 (CA125), cytokeratin-19 fragment (CYFRA 21-1), and carcinoembryonic antigen (CEA) are useful for the diagnosis of lung cancer^127–129^. However, data on these markers are limited, and their diagnostic efficacy is not high. Li et al. have proposed a 13-protein blood-based classifier with a negative predictive value of 90%^130^. A panel including 3 serum proteins (CEA, CA125, and CYFRA 21-1) and an AAb has shown 71% sensitivity and 88% specificity for lung cancer in a high-risk population^131^. Lower sensitivity (49%) but higher specificity (96%) has been reported in an independent validation cohort^132^. A four-marker protein panel (4MP) containing the precursor form of surfactant protein B, CA125, CEA, and CYFRA 21-1 has resulted in an area under the receiver operating characteristic curve of 0.79 (95% CI, 0.77–0.82). In combination with a risk prediction model (PLCOm2012), the sensitivity of 4MP can be substantially improved, by 11.9%^133^.

#### Volatile organic compounds

Volatile organic compounds are organic compounds with a high vapor pressure at room temperature, which show distinct patterns according to pathological state and are affected by the modification of proteins in various cellular processes. After production, volatile organic compounds are excreted into the blood, enter the lungs, and are exhaled^134^, at which point they can be tested and analyzed. Breath condensate analysis can be used to evaluate the presence of aldehydes, peroxide, leukotriene, cytokines, and adenosine, which are essential biomarkers of several different diseases, including lung cancer^135^. A previous study has reported a sensitivity of 94.2% and a specificity of 49.0% in lung cancer diagnosis based on exhaled-breath data combined with clinical parameters^136^. Although the diagnostic accuracy of exhaled-breath analysis for lung cancer has shown potential, a consensus is lacking regarding the normal reference range.

#### Other potential sources of biomarkers

DNA polymorphisms^137^, chromosomal abnormalities^138^, tumor-educated platelets^139,140^, extracellular vesicles^141^, bacterial biomarkers^142^, complement fragments^143^, urine metabolites^144^, and cytological analytes^145^ have also been proposed as potential biomarkers for lung cancer screening. However, the diagnostic potential of these biomarkers for lung cancer must be further evaluated, because the prospective evidence is limited. Combining these potential clinical biomarkers with radiological features, machine learning, and predictive models may improve the detection in screening of asymptomatic high-risk patients and help discriminate between benign and malignant nodules in the future.

### Exploration of treatment strategies for suspected malignancy

Guidelines and consensus statements have been released for the diagnosis and management of pulmonary nodules as the positivity rate of lung cancer screening increases rapidly. To date, surgery remains the best strategy for patients with strong clinical suspicion for stage I or II lung cancer^146^. The non-intubated approach is a promising thoracic procedure for GGNs and early-stage lung cancer, providing a safe, beneficial, less invasive alternative to intubated VATS, and enabling significantly shorter hospital stays^147^. During the spontaneous ventilation video-assisted thoracic surgery (SV-VATS) procedure, intraoperative intubation, post-operative chest tube placement, and urinary catheterization are avoided, thereby simplifying the surgical steps, decreasing patients’ post-operative discomfort, and enabling faster recovery^148^.

However, patients may be ineligible for surgery because of comorbidities, poor physical conditions, or poor pulmonary function^149^. Hence, identifying alternative strategies is crucial. In contrast to conventional radiotherapy, stereotactic body radiation therapy (SBRT) has become the predominant local therapeutic alternative to surgical resection. A pooled analysis of 2 early-terminated prospective trials comparing SBRT with surgery has reported promising results, including 15% greater OS with SBRT^150^. In addition, SBRT has been found to perform well in multiple primary lung cancers (MPLCs)^151,152^. Moreover, combing SBRT and PD-1/PD-L1 inhibitors has shown promise in early-stage NSCLC^153^. Thermal ablation (TA) was introduced to manage pulmonary nodules around the year of 2000. As a stand-alone technique, TA uses single or multiple percutaneous needles to deliver energy that heats or freezes the target area, thus causing necrosis of the target tissue^154^. Yamauchi et al.^155^ have conducted the first analysis focusing on cryoablation in patients with medically inoperable stage I NSCLC with tumor sizes mostly less than 2 cm, and reported OS rates of 88% at 2 years and 88% at 3 years. Although TA shows promise, some limitations exist. Contraindications include severe underlying interstitial lung diseases, such as pulmonary fibrosis, severe emphysema, and tumors less than 1 cm from the hilum^156^.

Beyond the treatments described above, the possibility of using agents to cure early-stage lung cancer is under investigation. With the adoption of lung cancer screening programs, patients with MPLCs are becoming a growing population in clinical practice worldwide^157^. Post-operative EGFR-TKIs have been found to have efficacy in unresected persistent GGO lesions in patients with MPLC who underwent resection of at least one EGFR-mutated lesion^158^. This observation has also been validated in KRAS-mutated lesions. Targeted therapy has therefore been suggested to serve as an alternative approach to treat MPLCs^159^. Moreover, a phase II trial has confirmed that sintilimab, an antibody to PD-1, has immune-related antitumor activity in GGO-featured lung cancer and is well tolerated among patients with early stage lung cancer^160^.

### Challenges in lung cancer screening and future directions

Although lung cancer screening through LDCT has meaningful clinical utility, broad concerns regarding its harm to patients have been raised, including false-positive screens, overdiagnosis, and radiation exposure. More than half of all pulmonary nodules are small, benign, and noncalcified^161^. Identifying cancers from false-positive results and preventing overdiagnosis in screening are challenging. NLST has set a positive cut-off threshold of at least 4 mm for any noncalcified nodule, thus leading to a 28.7% false-positive screening rate^162^. Patients may undergo unnecessary invasive examination and treatment because of overdiagnosis. According to an observational study, the LDCT overdiagnosis rate is 13%–27%^163,164^. Given the low specificity of LDCT, this method is currently considered to distinguish benign from malignant lesions through regular follow-up or in combination with other examination methods. Paci et al. have reported a low rate of overdiagnosis after an adequate follow-up period, thus indicating that overdiagnosis can be decreased through adherence to nodule management protocols^165^. The radiation exposure associated with screening is a matter of concern. The cumulative radiation from repeated CT scanning may independently increase the risk of radiation-related cancer^166^. However, some studies have indicated that the radiation of <100 mSv is negligible to the human body. In a secondary analysis based on an LDCT screening program for asymptomatic high-risk smokers 50 years of age or older who were scanned at least once per year^167^, the estimated median cumulative effective radiation dose after 10 years of screening was 13.0 mSv for women and 9.3 mSv for men^168^. In addition, other critical clinical questions presented in this review, such as risk-based selection of high-risk patients, nodule management, screening intervals, screening duration, and the standard for diagnosis and identification of malignant lesions, remain to be answered in the future.

## Conclusions

Screening with LDCT provides an opportunity to decrease mortality from lung cancer. With the identification of risk factors and optimization of entry criteria, the application of methods with high diagnostic accuracy, and the development of therapeutic strategies, the clinical benefits of early screening and diagnosis in people with lung cancer will further improve. Simultaneously, liquid biopsies are receiving substantial attention in cancer diagnosis as non-invasive, accurate, and predictive tools. These developments together promote the exploration and development of treatment strategies for detected and suspected malignancies, with the aim of optimizing clinical management and improving the prognosis of cancer patients.
